# On Coase and COVID-19

**DOI:** 10.1007/s10657-022-09741-w

**Published:** 2022-05-19

**Authors:** Darcy W. E. Allen, Chris Berg, Sinclair Davidson, Jason Potts

**Affiliations:** RMIT University, Carlton, Australia

**Keywords:** COVID-19, Coase theorem, Externality, Economics of pandemics, Comparative institutional economics, B52, D62, H23

## Abstract

From an epidemiological perspective, the COVID-19 pandemic is a public health crisis. From an economic perspective, it is an externality and a social cost. Strikingly, almost all economic policy to address the infection externality has been formulated within a Pigovian analysis of implicit taxes and subsidies directed by a social planner drawing on social cost-benefit analysis. In this paper we examine the alternative economic methodology of the externality. We seek to understand how an exchange-focused and institutional analysis provides a better understanding of how to minimise social cost. Our Coasean framework allows us to further develop a comparative institutional analysis of the pandemic response.

## Introduction

Government responses to the COVID-19 public health pandemic have rested on the notion that governments can intervene to mitigate the externalities of the virus. The dominant policy response has been to impose ‘social distancing’ on much of the economy to mitigate transmission externalities. Recent literature argues that those externalities might be less prevalent and less costly than otherwise assumed (see Leeson & Rouanet, 2021) and that we must consider the realistic information and incentive assumptions that underpin government responses to those externalities (see Coyne et al., 2021; Powell, 2021). Our contribution in this paper is, like that of Ronald Coase’s work, a methodological one. We explore and contrast the standard Pigovian analysis of the pandemic with a Coasean and comparative institutional approach (focused on bargaining and exchange over externalities).

Many economists responded to the COVID-19 pandemic by seeking a unified epidemiological-economic analytic framework that might yield insight into optimal lockdown policies. Economists combined the canonical susceptible-infected-recovered (SIR) epidemic model of the dynamics of a virus through a population with canonical macroeconomic models, including Dynamic Stochastic General Equilibrium (DSGE) models (Alvarez et al., 2020; Eichenbaum et al., 2020; Gonzalez-Eiras & Niepelt, 2020) with extensions to learning-by-doing models (Jones et al., 2020) and search-and-match models (Garibaldi et al., 2020). This family of unified SIR-DSGE models show that the decentralized equilibrium is inefficient because of the contagion externality and why a social welfare maximizing social planner will front-load mitigation and favour an earlier and harder lockdown than would decentralized agents.

A major limitation of this social planner perspective, however, is that the health shock and policy response are both assumed to be exogenous. This is particularly the case in epidemiological models that tend to use historical data to calibrate agent behaviour, rather than assuming agents will respond to incentives or themselves form rational expectations-type models to guide their decentralised actions. In a Lucas-critique style analysis, Chang and Velasco (2020) develop an economic theory of pandemics with forward-looking agents that shows how individual decisions about whether to go to work affect transmission dynamics, yet these decisions are endogenous to economic expectations of policy actions that affect the consequences of going to work (e.g. trust in government, expected stimulus, expected behaviour of other agents given the expected stimulus, etc.). The extent of endogeneity in externalities and expectations and the complex feedback between public health diagnostics and economic policy treatments has been a major revelation as both public health experts and economic policy-makers have scrambled to deal with the COVID-19 pandemic (see also Born et al., 2021).

Another limitation of the social planner models is their reliance on representative-agent or uniform models. They treat populations and the policies imparted on them as homogenous. This simplified population analysis is largely for modelling convenience and due to severe real time data limitations. Elaborations of the unified SIR-DSGE social choice models have introduced targeted-versus-uniform lockdown policy in a multi-risk model, reporting “the qualitative finding that semi-targeted policies significantly outperform uniform policies” (Acemoglu et al., 2020, p. 4). In that model, social welfare was maximized with targeted policies that focused on protecting high-risk subpopulations rather than the uniform policies favoured by epidemiologists. This analysis is interesting because it shows how in a pandemic context a social planner can trade-off the costs and benefits to different groups in order to maximize a social welfare function. Social planners internalise externalities through taxation of work and consumption to affect containment (the stick) and redistribution to subsidise containment (the carrot).

This conception of social distancing is particularly Pigovian, where a mandatory social distancing policy is the equivalent of a 100 per cent tax rate on that activity. This approach forms the dominant modelling assumption so far used in analysis of optimal policy response to COVID-19. But once we start thinking about pandemics from an economic policy perspective, and then extend that to multiple agents, a new analytic framework comes into view: a Coasean analysis built around Ronald Coase’s famous theorem (Coase, 1959, 1960). A Coasean approach to the pandemic variously focuses on the reciprocal nature of externalities and the institutional conditions under which those externalities may or may not be bargained away through exchange (e.g. see Williamson, 2020; Coyne et al., 2021; Leeson & Rouanet, 2021; Boettke & Powell, 2021; Paniagua & Rayamajhee, 2021). This paper builds these insights together with comparative institutional analysis to explore and understand public policy responses to the pandemic.

In the Pigovian model of externalities, a social planner intervenes to reallocate economic resources in order to internalise the externality (in this case the contagion and congestion externality, Jones et al., 2020). In the Coasean approach, parties bargain their way to a solution that is resolved with an exchange that internalizes the externality. In the uniform SIR-DSGE model there is no possibility of Coasean bargaining because there is effectively just one agent (the susceptible population) who then experience probabilities of transitions to states of infection and recovery. It is the social planner who chooses in this formulation, and the policy choices then update the parameters of the model society.

In a model with individual decentralized agents, each agent’s choices impose externalities on others. These impositions are propagated unevenly and fall unevenly across the economy. When someone decides to go to leave their home when infected they may be incentivized to do so for leisure or other economic benefits, but there is no way for those who are harmed by that action (by increasing the risk of infection for others) to offer an incentive to stay home. There are missing property rights and missing markets to enable all the third parties to pay them to stay at home. It is important to note here that, as argued in Leeson and Rouanet (2021, p. 1113), the externalities imposed on private property are of a different nature to that on public property: “residual infection risk that visitors face from the on-site behaviours of other visitors is infection risk that they face contractually and thus risk that does not impose on-site external costs” (see also Boetkke & Powell, 2021). That is, externalities on private property, such as on-site transmissions that occur at a café, are internalized through contracts with higher or lower prices. Nevertheless, in economic theory the transaction costs of each agent contracting with others to stay at home, or not go shopping, would swamp the expected benefits of the trade. It would simply be too costly for those mutually beneficial trades to be discovered, negotiated and enforced. So a decentralised model will not be able to internalise the externality through trade. This is why the unified SIR-DSGE models recognize the existence of the COVID-19 externality and that social welfare suboptimality of a decentralized solution.

Now consider the Coasean analysis between coalitions sorted by risk, as in the multi-risk Acemoglu et al. (2020) model.^1^ Suppose for simplicity there are just two self-identified and self-sorting groups: (1) high risk of death from the disease; and (2) low risk of death. (We consider below the implications of strategic deception about identity in these groups, and the effects of uncertainty.) Around the world, governments have everywhere adopted a uniform lockdown policy, regardless of cohort risk. This has in effect imposed different statistical costs and benefits on the different risk groups. The low-risk groups are paying a large price in terms of lost utility from work and consumption to benefit a different group in terms of changed risk of mortality. But these are statistical not absolute risk groups. A uniform policy means that governments do not need to identify, target or differentially reinforce policies: they apply to all citizens. Nevertheless, as the Acemoglu et al. (2020) analysis indicates, if the information and enforcement cost assumptions of the model are true, a targeted approach would be superior in terms of minimizing deaths and economic losses.

A Coasean analysis asks a different question: could the groups themselves bargain their way to the same Pareto superior equilibrium? Or is a social planner necessary to get to good equilibria? To address this, we need to think about which group is imposing externalities on whom. If the low-risk group is freely moving about then they are imposing a contagion externality on the high-risk group. This is due to a lower expected cost of infection, i.e. the low-risk group expects to recover and not to die. But if the high-risk group prohibits the low-risk group from moving freely about and making a living, then the externality is being imposed by the high-risk group on the low-risk group. (There is also a congestion externality that is imposed inter-group, as each person who goes to work or to market imposes an increased risk on everyone else who also decides to go, but we ignore that here.) Just as in Coase (1960), both groups are imposing costs on the other groups—that is, the externalities are reciprocal. The Coasean analysis asks not who is causing the harm (a question perhaps of morality or justice), but rather who can avoid the harm at lowest cost (a question of economic efficiency). Under institutional conditions of clear property rights and low transaction costs we expect that a bargaining solution to arrive at the most economically efficient solution (see McChesney, 2006; Fox, 2007). Indeed, as Boettke and Powell (2021, p. 1095) describe in the standard law and economics approach this would involve “assigning rights such that the least cost mitigator bears the burden of adjusting to the externality.”

Whether such conditions hold depends upon a range of cultural, social and political factors. For instance, the high-risk groups (typically those over 65) may bargain with low-risk groups (working age populations) through political-democratic brokering. For instance, they might send messages to politicians that use voting blocs as rewards or threats. High risk groups could use political power to force a uniform policy. In turn low-risk groups may agree to that bargain in return for expected wealth transfers through raids on fixed income pension commitments through inflation, etc. As such, we can think of the policy decision not through the additive utility lens of a social welfare function (a Pigovian lens) but rather as political brokering of coalitional exchanges across different risk groups in society (a Coasean lens). In this way we can shift from what James Buchanan (1964) referred to as an ‘allocation’ lens of economic inquiry towards an ‘exchange’ paradigm. The former emphasizes the allocation of resources by a social planner with relevant information, while the latter “focuses on the process of interaction between people within a context-specific, and varying, institutional environments”, with more realistic assumptions about information and incentives (Coyne et al., 2021, p. 1122).

If transaction costs were zero, then “…each person would strike a deal with every other person whose infection risk their behavior might affect or whose behavior might affect their infection risk. All costs of such behavior would be internalised” (Leeson & Rouanet, 2021, p. 1109). Of course, even that simple two-group politically-mediated Coasean bargaining prospect is conditional upon secure property rights (in this case well-formed and credible voting coalitions) as well as good and easily identifiable information about which risk group each individual belongs to. As further groups are identified the epistemic challenges compound. As Coyne et al., (2021, p. 1119) explore, the political economy of state responses to COVID-19 must consider both policymakers’ “epistemic constraints they face in trying to solve that problem” as well as those policymakers’ incentives. As Williamson (2020) argues in developing a Coasean social contract model of the pandemic, the “… large variations in individual trade‐offs and private information about such trade‐offs” suggests solutions based on individual choices and incentives rather than mandates. Early in the pandemic, however, there was a great deal of ambiguity about information such as risk profiles, and while growing evidence does seem to confirm that specific factors do characterise distinct risk groups (e.g. age, comorbidity) significant epidemiological uncertainty remains. Nevertheless, even a simple or ‘naive Coasean’ analysis of the exchange approach to COVID-19 policy is likely to yield valuable insight to address important economic issues and considerations that are largely or entirely ignored by the Pigovian or social welfare economic analysis.

A public health crisis involving an infectious disease is clearly a negative externality. Those infected individuals encountering non-infected healthy individuals can pass on the disease to those individuals resulting in their subsequent illness, or even death. In the very first instance, this can be described as being a ‘health externality’, which are typically negative.^2^ Further distinctions between the type of externalities in a pandemic have been outlined, such as ‘on-site externalities’ (where people impose externalities on others at a given site) and ‘off-site externalities’ (where the externalities are the effect of increasing infection risk of others at different sites) (see Leeson & Rouanet, 2021). Further complicating the nature of pandemic externalities, Rayamajhee et al. (2021) argue that pandemic externalities, rather than being global as is often assumed, are in reality “nested externalities at multiple scales”, where different actions at different scales have costs or benefits. For our purposes, we distinguish simply between a ‘health externality’ and a ‘behavioural externality’.

One of the challenges facing decision makers in relation to the COVID-19 pandemic is the lack of information associated with the virus itself, including the ‘health externality’ associated with it. Initially there was no knowledge of the characteristics of the virus—how much time there was between infection and symptoms, how contagious it might be and under what circumstances, what the fatality rate was, and so on. The social cost associated with spreading COVID-19 was unknown or highly uncertain through February and March 2020 when policy choices were being made. For the most part, policymakers in most countries assumed the social cost would be very high, and the unprecedented global policy responses (relative to viruses such as the seasonal flu) reflect that assumption.

The second externality (the ‘behavioural externality’) caused by COVID-19 may be either negative or positive. The behavioural response to the pandemic resulted in individuals *voluntarily* self-isolating in order to prevent themselves from contracting COVID-19. This ‘behavioural externality’ has similarities to a pecuniary externality in a market. To the extent that individuals withdraw from economic activity and reduce their consumption, this imposes costs on others and is a negative externality. It could also be the case, however, that these individuals, by following their own self-interest, inhibit the spread of the virus. If this were the case, then their behavioural response is a positive externality. On balance, the net externality could be positive or negative. For reasons that we explain below, policy makers acted in a way that suggests the net effect of this behavioural externality to be negative.

While previous studies have examined the nature of externalities in the pandemic (e.g. Leeson & Rouanet, 2021; Rayamajhee et al., 2021), the political economy of state responses (e.g. Boettke & Powell, 2021; Coyne et al., 2021), the complexity of pandemic policy (e.g. Pennington, 2021) and the economic consequences of the pandemic (Allen et al., 2020), this paper draws theoretical insights from Coasean economic theory, integrating these findings into a broader comparative institutional and public choice perspective. In Sect. 2 we discuss the origin and various meanings of the Coase theorem. In Sect. 3 we apply these to the COVID-19 pandemic using the framework of comparative institutional analysis. Section 4 considers pandemic management as a transaction cost problem. Section 5 examines some public choice theory considerations. Conclusions are offered in Sect. 6.

## Beyond vulgar Coaseanism

One challenge for economists when approaching the Coase theorem is that there are many interpretations of what that theorem might be. Some economists, like Paul Samuelson, suggested that the Coase theorem was not a theorem at all. Other economists, like George Stigler, conflated it with other insights (on Stigler and Coase see Marciano, 2018). Unfortunately, Ronald Coase himself gave some credence to the Stigler interpretation, while insisting that he had made more of a methodological contribution as opposed to a hard and fast insight.

Coase (1988, p. 157) reports that he first expressed his theorem in a 1959 paper that had appeared in the *Journal of Law and Economics*. There he had made use of the example of a newly discovered cave. The argument was that the initial ownership of the cave and the ultimate use of the cave were independent of each other. The cave would be put to its most valuable use. The more famous 1960 article was an elaboration of that principle. As Coase (1988) notes, in the absence of transaction costs there can be no deviation between private and social costs.

Deirdre McCloskey (1988, p. 368) argues that this version of the Coase theorem is really ‘Adam Smith’s theorem’—that resources will gravitate into the hands of those who value them the most—if transaction costs are zero. According to McCloskey, the Coase theorem tells us that transaction costs do matter. While this is correct, it does seem to abstract from Coase’s other important contributions in his 1960 paper. Rather than express a theorem, Coase was attempting to make a methodological point—that how economists thought about social cost suffered from basic defects.

Coase recognised and emphasised that social costs problems (i.e. externalities) were reciprocal. In many of his examples the individuals were imposing harm upon each other. The question in Coase’s mind was: who should harm whom? The answer that he kept returning to was the arrangement which maximised the value of production. By contrast, the Pigovian solution to externalities would be to determine who had injured whom, and then require the advantaged party to compensate the injured party or levy a tax on the advantaged party.

Coase (1960, p. 131) also suggested, unkindly but not incorrectly, that economists did not carefully think through the problems at hand, leading them “to declaim about the disadvantages of private enterprise and the need for Government regulation.” He also argued that economists did not explore the full set of possible solutions to any problem of social cost. When confronted by a social cost, rather than immediately consider government regulation, there are other solutions that should be carefully evaluated. The immediate and obvious solution to any problem, he argued, is to do nothing—arguing that very often the costs of doing something would be greater than the benefits of that action. Then he suggests that markets could be deployed to resolve social costs. In a world of zero transaction costs the Adam Smith principle applies. Coase (1960), however, recognised that transaction costs may not be zero, or even low, and that markets would not always be able to resolve negative externalities.

It is important to dwell on that point. Very often Coasean solutions to externalities suggest that all that needs to be done is for property rights to be allocated to a party and then leave the market to allocate use rights. This may be *a* Coasean solution to the problem of social cost, but it is not *the* Coasean solution. This ‘let winners compensate losers’ or ‘losers bribe winners’ approach to resolving problems of social costs can be described as ‘vulgar Coaseanism’.

In the presence of market failure, given transaction costs, Coase (1960, p. 115) points to his 1937 paper on the nature of firm. There he had argued that hierarchical costs within the firm could be lower than transaction costs within the market and that firms existed when administrative decision-making costs were lower than market transaction costs. It is possible that some social costs can be privatised through vertical integration.

Only after the relative costs and benefits of doing nothing, relying on market forces, and vertical integration were considered, should government intervention be considered. Importantly Coase suggests that government intervention—Pigovian solutions—have costs and benefits and may fail to resolve problems of social cost just as markets do. Coase suggests that government intervention is likely to be more effective when coordination costs are high. Government does not need to incur the same coordination costs as do private actors—it can simply deploy its police power to impose solutions, including in situations where “… a large number of people are involved and in which therefore the costs of handling the problem through the market or the firm may be high” (Coase 1960, p. 118).

Coase’s (1960) contribution was not to demonstrate that transaction costs do or do not matter, or that market solutions require property rights, or that government intervention can fail too. His contribution was that economists should think carefully about potential solutions to the problem of social cost and evaluate real world alternatives. Indeed, “satisfactory views on policy can only come from a patient study of how, in practice, the market, firms and governments handle the problem of harmful effects.” (Coase 1960: 118). Harold Demsetz (1969) described decision making based on blackboard economics as being ‘nirvana economics’, which advocates a comparison between an idealized alternative and a real-world alternative. Coase advocates what Demsetz (1969) labels as being ‘comparative institutional’ analysis between real world alternatives. We apply such an approach to the COVID-19 policy responses in the following sections.

## An institutional choice framework for the COVID-19 pandemic

To understand the various responses to the externalities generated by the COVID-19 pandemic, we draw on this Coasean lens and combine it with the institutional possibilities frontier framework first proposed by Djankov et al. (2003). Djankov et al. (2003) were interested in explaining the growth of regulation over the course of the twentieth century and to explain why regulation seemed more prevalent in high-income economies. The frontier itself traces the trade-off between (private) disorder costs and (public) dictatorship costs. Following the Coasean insight, the costs of using market-based regulatory mechanisms are traded-off against the costs of using government-based regulatory mechanisms. In this context, disorder is defined as being “… the risk to individuals and their property of private expropriation in such forms as banditry, murder, theft, violation of agreements, torts, or monopoly pricing” (Djankov et al., 2003, p. 598). Dictatorship is defined as being “… the risk to individuals and their property of expropriation by the state and its agents in such forms as murder, taxation, or violation of property” (Djankov et al., 2003, p. 598).

Djankov et al. (2003) then use this framework to examine four broad governance strategies that can be used to achieve some regulatory objective: ‘market discipline’, ‘private litigation’, ‘public regulatory enforcement’, and ‘state ownership’. In the analysis that follows we define disorder costs as the negative externality imposed on other individuals due to infection and a voluntary behavioural response to the pandemic. Dictatorship costs are the costs imposed by the government in response to the pandemic such as enforcement of quarantine, loss of civil liberties, and the like. Dictatorship costs include loss of economic opportunity that results from quarantine policies. It does not, however, include the costs of ‘hibernating’ the economy and costs incurred in restarting the economy after the quarantine period ends.

With that background, it is possible to set out a series of responses and policy approaches to the COVID-19 pandemic. For the sake of completeness, we include a ‘Do-nothing’ response. In this response, nobody does anything in response to the pandemic. Individuals do not modify their behaviour in any way, nor do governments respond in any way. Under this response, individuals go about their lives and infect other individuals. Currently the medical understanding of COVID-19 is that some infected individuals will not develop any symptoms of the disease and will not feel unwell at all. Asymptomatic individuals may still be infectious. Other infected individuals will become ill but will recover. Yet others will become very ill, and some will die. In this response the disorder costs are very high. The virus simply transmits through the population and the costs associated with the health externality are maximised. An epidemiological model of this process can be calibrated with the standard SIR model.

This ‘Do-nothing’ scenario is extremely unlikely and did not occur. Individuals respond to medical crises. For example, individuals who become ill may take sick-leave from work. Those individuals who are vulnerable to infection may self-isolate. Others may withdraw their children from school or stop visiting crowded places such as cinemas, clubs, gyms, and the like. This scenario we label as ‘voluntary individual self-isolation’. In this response we see some reduction in the costs due to a health externality, but the introduction of a behavioural net negative externality. Some service providers may experience financial loss due to consumers reducing their purchases and changes to consumer behaviour. Related to this, Leeson and Rouanet (2021) argue that the externality context of COVID-19 suggests that the externalities are somewhat self-limiting.

The next response level we describe as being ‘voluntary corporate self-isolation’. Employers may voluntarily reduce the scale of their operations or even cease operations to protect their staff. This could entail reduced working hours, or fewer staff working during each shift. Schools could adopt distance learning models and some employees could work from home. Note that this is distinct from government mandating employers to restrict their staff, which occurred in many jurisdictions.

The responses we have described so far are voluntary. The social costs that are being imposed are disorder costs. Government may have provided public information and/or made recommendations in the scenarios and responses that we have described, but as yet there are no dictatorship costs in the composition of social costs being incurred. What is important to note is that as each scenario has emerged that the social costs due to the health externality are likely to be falling, while the social costs due to the behavioural externality are likely to be rising. The behavioural externality will result in disorder costs such as reduced amounts of economic activity resulting in job losses. It could (and did) result in panic buying and hoarding. Many countries experienced toilet paper shortages for example—prior to the imposition of formal and mandatory quotas. Very few governments appear to have relied on a voluntary response to the pandemic—Sweden and some Swiss cantons appear to have adopted this approach, while the United Kingdom initially indicated that it would adopt a voluntary approach to the pandemic it quickly changed tack.

Government responses to the COVID-19 pandemic have focussed on the health externality. Individual responses that were based on voluntary self-isolation were transformed by government fiat into involuntary quarantine policies. For the purposes of illustration three versions of involuntary quarantine policy can be described. *Mild quarantine* consists of the government requiring that most people stay at home with only essential workers going to work. Essential workers here can be broadly defined. Under mild quarantine individuals might be allowed out of their homes for shopping and exercise at their own discretion. The police, however, do enforce the quarantine and do issue fines for quarantine violation. *Strict quarantine* consists of more restrictive definitions of essential workers and fewer exemptions to home quarantine. Individuals may be restricted on what they may buy (e.g. some countries have closed non-essential retail stores) or when they may leave their homes (e.g. only one adult may leave the home every three days, or an overnight curfew). *Absolute quarantine*—also included for completeness—is a situation where no-one is permitted to leave their homes for any reason. This form of quarantine is viable for very short periods of time only.

In these scenarios the behavioural externality that previously existed is now replaced with a dictatorship cost. Those individuals who would have self-isolated anyway under the same conditions as the government imposes are no better or worse off than they were before. Those individuals who would have self-isolated to a lesser extent or not at all are worse off than they were.

The important question, however, is which response or scenario results in a minimisation of social costs (from both disorder and dictatorship).

The first point to make is that the existence of a negative externality is not itself a necessary and sufficient condition for a response. As Coase pointed out, doing nothing is an option. As we know, however, people do not do nothing in the face of a medical emergency. There is a response—people both self-isolate and change their consumption and productive behaviour. For there to be a justification for policy intervention an externality must persist in equilibrium (Buchanan & Stubblebine, 1962). In disequilibrium social costs and private costs may diverge from each other. As externalities are internalised due to behavioural responses the divergence between social and private costs will fall. If that differential falls to zero before equilibrium, then there is no market failure. In the Buchanan and Stubblebine terminology the externality is not Pareto relevant. It may be the case, however, that the externality persists in equilibrium—that is, it is Pareto relevant. At that point market failure has occurred.

In the case of COVID-19 market failure occurs when individuals, despite their voluntary behavioural responses, are still imposing costs upon each other. Given that there are two externalities at work, this is very likely to be the case. The health externality and the behavioural externality work in opposite directions to each other. As more people choose to voluntarily self-isolate to avoid contracting the virus, they impose greater behavioural costs on others.

## Pandemic management as a transaction cost problem

Setting out the institutional choices in such a way requires us to ask several uncomfortable and unavoidable questions. What is the optimal rate of infection from a public policy perspective? How does that compare to a private perspective? Arrow’s impossibility theorem indicates that the policy choice made by the government is not going to be some aggregate of private preferences.

Most developed world governments have sought to slow the rate of infections to target medical capacity to deal with the pandemic. This is the ‘flatten the curve’ model, most influential in March 2020 when many governments were making their institutional choices, in which the total number of individuals who are eventually infected is fixed (see Allen et al., 2020). The goal of flattening the curve is spacing infections through time to prevent a sudden influx of COVID-19 cases from over-burdening health care systems.

The choice of this objective function introduces another consideration into the debate: individuals are not just imposing costs upon each other; they are imposing a cost on the health system. This healthcare system may or may not be public, or have complex public/private entanglements (on entanglement see Smith et al., 2011; Wagner, 2016). It may well be the case that no externality exists in equilibrium from a health perspective *but for the health system*. This insight is a law and economics, or public choice, problem and we defer discussion to a later section.

Irrespective of why governments chose a particular objective function the net effect of intervention was to assume that a health externality persisted in equilibrium, and to substitute private disorder costs due to the behavioural response to the pandemic with dictatorship costs. This cannot be an equilibrium solution. Government intervention in response to externality is to restore an equilibrium situation that would exist but for the shock to the equilibrium.

Market failure is usually due to one of four factors: monopoly problems, missing markets, asymmetric information, or transaction costs. The COVID-19 pandemic is not obviously a monopoly problem. It would be too easy and glib to suggest that the COVID-19 market failure was due to missing markets. To suggest that individuals vulnerable to the virus be given property rights to their continued health and be paid to self-isolate would be a ‘vulgar Coasean’ solution. So too the notion that vulnerable individuals pay everyone else to remain in quarantine.

It is useful, however, to think about ‘rights’ that individuals may have in the face of a pandemic. Vulnerable people can suggest that they have a right to life that in this instance includes the right to not become infected. Others may argue that they have a right to a livelihood or a right to choose. Reconciling competing rights, especially in the absence of cash payments, is difficult at the best of times. It does, however, go the question of who should be quarantined—just the vulnerable, or everyone? Almost uniformly governments have chosen to quarantine everyone (although in many jurisdictions vulnerable people have been subject to stricter rules, such as restrictions on access to aged care). Garzaerlli et al. (2022, p. 1) explores this uniformity through the lens of Rawlsianism, arguing that “lockdown by fiat is a policy that is closer to a maximin equity criterion rather than to a utilitarian one”.

What many governments have also chosen to do is make payments to those individuals who have either lost their jobs (beyond the usual unemployment benefit that might normally be paid under such situations) or have been temporarily stood down or furloughed. Details vary across jurisdictions, but the principle is broadly similar—employers who have been impacted by the quarantine policy can apply for a wage subsidy to be paid to their employees. This may strike some readers as being a Coasean payment for lost wages. But this money is not being transferred from winners to losers. Instead, it is being transferred through time, from future generations to current generations. The money is being borrowed (or printed—the macroeconomic consequences of the quarantine policy will be debated for decades and is beyond the scope of this article) and will be repaid from future tax revenues or budget cuts or inflation.

It is likely that a market failure exists due to transaction cost problems and information cost problems. A lack of information problem is distinct from an asymmetric information problem. Asymmetric information is possible but not likely to be significant for this analysis. For example, it may be possible for an individual to be knowingly infected, or to believe they are likely to be infected, but externally asymptomatic and infect others.

Before we proceed to discuss transaction cost and information cost problems, it is useful to point out that the market failure is not due to individuals simply being selfish. It is easy to argue that markets could simply fail to clear because individuals are selfish—the welfare of others simply does not enter into their utility function, and they are simply indifferent to other individuals’ premature or preventable death. This argument has been made by the authorities when justifying authoritarian regulation or enforcement of quarantine. The health externality is reciprocal—individuals may either infect others or become infected themselves. Indeed, Williamson (2020, p. 157) proposes a Coasean social contract model that “recognizes the reciprocal nature of the problem.” While the virus does tend to be more fatal to older and immuno-compromised individuals it is infectious and has a non-zero fatality rate for all humans. To the extent that individuals have no voluntary behavioural response to the COVID-19 pandemic this is very likely due to information asymmetry or direct economic incentive. Responding to direct economic incentives may be anti-social but it is not a market failure.

The direct cause of the market failure—assuming the market has failed—is the existence of radical uncertainty. Mainstream economics tends to make strong information assumptions to drive its results. When those assumptions are relaxed, they are so that information is costly (i.e. the information exists but must be acquired at a price) or is asymmetrically distributed. One of the features of the COVID-19 pandemic is that information either did not exist, or was highly uncertain, or contested. Behaviour must be conditioned by expectations which in turn is conditioned upon information. Bounded rationality—first proposed by Herbert Simon and popularised by Oliver Williamson (1985)—results in individuals making and using heuristics, rules of thumb, and various mental short-cuts when decision-making.

In the months that the COVID-19 disease emerged and spread globally, there was a high degree of this sort of radical uncertainty, around almost all epidemiologically relevant aspects of the disease. It is possible that individuals under-estimated the COVID-19 infection rate or severity and subsequently self-isolated too little, resulting in a health social cost in equilibrium. It is also possible that the government over-estimated the COVID-19 infection rate and imposed high dictatorship costs on the economy when there was no social cost in equilibrium. Given the breadth of these uncertainties and the sensitivity of comparative institutional analysis to those uncertain factors, it is implausible to suggest that the policy choices made between February and March 2020 were anywhere approaching optimal.

But epistemic issues facing the epidemiology of the virus itself are only the “first layer of complexity that policymakers must contend with” (Pennington, 2021, p. 204). Drawing on Hayek’s distinction between simple and complex phenomena, Pennington notes that even while government action might be warranted in response to the externalities, the complex nature of the problem means that determining an effective policy response is difficult. Indeed, the health effects of the virus spread are interacting with further complex phenomena of “political, economic, cultural and institutional arrangements” (Pennington, 2021, p. 208).

Radical uncertainty, however, does not directly explain why different governments imposed various degrees of strictness on quarantine conditions. While information about COVID-19 was uncertain, and the medical science preliminary, given the extraordinary effort made by public authorities and researchers around the world to investigate the characteristics of the disease, the information was highly accessible. International coordinating organisations, such as the World Health Organisation, also sought to provide governments with consistent responses.

One explanation of differing policies is different levels of ‘trust’ or ‘civic capital’ in different jurisdictions, as identified in the Djankov et al. (2003) framework. For instance, jurisdictions with higher levels of ‘civic capital’ might (1) be more confident that populations will voluntarily comply; and (2) have less tolerance of high dictatorship costs because of their democratic ideals, leading them to have fewer restrictions. As Rayamajhee et al. (2021, p. 12) argues, because ‘social distancing’ is co-produced between citizens and governments, “a provincial or national authority with a history of betraying public trust is unlikely to effectively implement social distancing guidelines/policies”. Related to this, Paniague and Rayamajhee (2021) draw on Elinor Ostrom’s work to frame the challenge of the pandemic as one of “nested externalities that are organized in multiple, overlapping scales”. This suggests the need for a polycentric approach to pandemic governance challenges, acknowledging the need for institutional diversity and flexibility in response (see also Allen et al., 2020).

The stark differences in cross-jurisdictional approaches can also be explained from an epistemic perspective. The various costs of dictatorship and disorder relating to pandemic policies are subjective. As Allen and Berg (2017) argue, societies that view the trade-offs between different regulatory regimes in different ways. Similarly focusing on the epistemic challenges of pandemic policies—with an emphasis on the complexity of the problem—Coyne et al. (2021) point to the need to match externalities with their lowest level of decision making. From this perspective, the different policy approaches across jurisdictions have some benefit, in revealing or discovering information about effective policy responses. This policy learning process, however, is limited by a “‘signal extraction problem’ in deciphering what the results of various policy experiments may mean and whether any lessons can be applied elsewhere” (Pennington, 2021, p. 213).

## The law and economics of the COVID-19 pandemic

Pigovian approaches to policy are made more fraught by the fact that the government is not a disinterested actor. Public choice theory—and law and economics more broadly—is the study of how government (and the politicians and bureaucrats that comprise it) is a distinct actor within an economic system with its own economic incentives (Mueller, 1976). One theoretical foundation is Arrow’s (1950, 1951) critique of social choice functions, which showed how it was impossible to aggregate private utility into a social utility function without violating some desirable conditions, one being the no dictatorship rule (i.e. that one agent’s preferences dominate all other agent’s preferences). Yet in the context of public policy to address COVID-19, exactly this situation has arisen in which the ‘dictator’s’ preferences for resource allocation may depart from the preferences of individual citizens, however aggregated.^3^ Note this does not depend on citizens having different preferences, and this wedge between the incentives of the state and the sum of incentives of citizens will hold even with identical preferences across all citizens.

As we introduced in Sect. 3 above, when individuals move from susceptible to infectious they are not just imposing costs upon each other as a contagion externality on private individuals, they are also imposing a cost on the health system, as an externality on the state. For instance, individual citizens will have private preferences not to become infected and to die from COVID-19, and these preferences will extend to social preferences^4^ for this fate to not befall others too. Governments, on the other hand, have preferences focused on the public health system, which they seek to protect, not on individual citizens. This is not a cynical point: the UK government, for instance, has directly explained this point in public communication, namely that the strategy was to protect their National Health System (NHS). The ‘flatten the curve’ diagrams were expressly designed, and communicated, as a strategy to protect the capacity of the public hospital systems. To put this succinctly, from the government’s perspective, they obviously do not want their citizens to die; but should they die, it is better that they should do so without harming treatment capacity in public hospitals. This is not a heartless statement, but an expression of the margin of concern for the government supplying public healthcare during a pandemic.

Another way of seeing this same point is to look at it from a dynamic planning perspective, recognising that it is extremely costly to ramp up or quickly substitute one type of health service for another due to asset specificity in medical equipment, hospitals, and skilled labour. Medical equipment cannot be quickly repurposed. At time t = 0, governments allocate funding X to public health on the assumption that it will need to provide services Y at t = 1. Any demand above Y at t = 1 (or a different configuration of demand) creates rationing, which is a cost borne by citizens. This is politically costly as those rationed citizens will punish the incumbent government because the excess demand signals that t = 0 government failed to properly plan for t = 1 scenarios.

But this sort of bureaucratic forward planning and budget allocations is destined to fail because of poor information and incentives by the bureaucratic agents (Mises, 1944), or worse, will be fully captured to pursue the agents own ends (Niskanen, 1971). Nevertheless, the legitimacy of the modern welfare system relies very heavily on the state delivering public goods to the population. Having the public health system collapse under the weight of a pandemic would be a major embarrassment to the perceptions of competence and legitimacy of a government.

Once a health budget has been allocated at t = 0 (including a spending level and an allocation across services) a government experiences what Williamson (1985) calls a ‘fundamental transformation’ where they no longer have a wide set of options going forward, but a narrow set of capabilities that can only deal with a predetermined range of events. Any events falling outside that planning window will overwhelm the health system, which means to blow out the budget. Governments will therefore be incentivised to order events so that they fall within the health sector capability, even when that means imposing an externality back on citizens by for instance shutting down all elective surgery or banning any activity that could place demand on the health system such as driving, sports. Similar incentives extend to preferences over subsidising employment (in Australia, called the JobKeeper program) to avoid overwhelming the unemployment provisions and budgets allocated to welfare.

The COVID-19 pandemic and the government response also raise further questions that can be analysed through law and economics (and public choice theory) that we flag here as topics for subsequent inquiry. Most broadly, as Boettke and Powell (2021, p. 1090) argue, we must examine the “incentives and information that confront policymakers and voters and the institutional environments that shape their incentives and information”. As Coyne et al (2021) point out, the nature of political competition can lead to rent seeking in response to a public health crisis, where individuals exert influence on the public policy process to allocate resources that benefit themselves.

First, following Downs’ (1957) theory of log-rolling and the economics of political parties, we predict that the urgency to enact legislation to address the pandemic will significantly lower bargaining costs associated with vote trading (Buchanan & Tullock, 1962), leading to an increased number of back-room deals being made in order for the party in power to be able to present a single coronavirus emergency response omnibus bill before parliament or congress for expedited approval during some manner or restricted debate or sitting period.

A further prediction is that the lowered bargaining costs, due to the higher opportunity cost of a failure to reach consensus and to deliver effective emergency measures legislation, is that the efficiency of a multi-party system is reduced, as there only effectively needs to be one party, with all special interests able to deal behind the scenes. In times of crisis there is a common tendency to rally-around-the-flag (Mueller, 1985) and to support incumbent leaders and their party. Knowing this, rational opposition parties will put effort into political bargaining (vote-trading or log-rolling) toward a consensus bill rather than seeking to present an alternative legislative agenda.

This collapse in multi-party competition driven by falling political bargaining costs (because of the emergency response) and resultant omnibus legislative bundle rushed through the political process (to economise on political costs), which will therefore be complex and far less scrutinized than in normal times, is then predicted to have a further behavioural effect that the legislative act will be difficult to understand by individual voters, who indeed will have no incentive to understand the details (they will be rationally ignorant, Caplan, 2007), but will also give rise to expressive voting (Buchanan & Brennan, 1984), or conspicuous signalling of support for the consensus bill, and using social mechanisms to enforce compliance (shaming in public or on media, rallies of support, expressions of anger and even violence). In the COVID-19 pandemic, this process of aggressive public consent soon targeted any counter-narrative of the value of opening the economy back up.

We could also consider the long run effect examined by Olson (1982) on the economies of Germany and Japan after the Second World War, in which one of the benefits of losing the war was the institutional destruction of rent seeking regulations and legislation and clearing away of thickets of special deals between Pre-War elites that had accumulated over long periods of peaceful prosperity, but were a significant drag on efficient competition and resource allocation. The urgent deregulation of unnecessary regulations and licencing regimes, particularly in relation to urgently needed production and innovation in health and other essential industries, provides the opportunity for a constitutional or institutional reset, following defeat. From this perspective, a pandemic may have similar effects on long run economic growth as losing a war due to the opportunities for institutional creative destruction.

## Conclusion

Negative externalities arising from an economic activity impose a social cost. This can be dealt with through government intervention targeting that activity—directly through regulation, or indirectly through market interventions (e.g. through taxation or subsidy to internalise the externality and minimise the social cost of an economic activity). This is called the Pigovian approach, after A.C. Pigou, who developed the foundations of modern welfare economics. But Ronald Coase recognised that this was not the only solution to the problem of social cost because it failed to recognise the symmetry in any situation of externalities, and therefore fails to focus on the problem of maximizing economic efficiency. Provided transaction costs are low and property rights are clear, Coase explained, parties can bargain their way to an efficient solution to externality problems.

We expect the coming months and years to feature heated retrospective debate about what policies were most effective in limiting the spread of COVID-19, and the relative trade-offs of those policies vis-a-vis their effect on economic activity. Much of that work will be empirical. But this paper has argued that there is a higher-level debate to be had about the policy framework that was adopted.

COVID-19 is a viral pandemic, causing a global public health crisis, but from an economic perspective it can be understood as a negative externality, thus presenting two policy pathways forward. To date, and globally, almost all public economic policy response has gone down the Pigovian path. However, from an economic theory perspective, there are arguments as to why a Coasean perspective could on some margins be a superior basis for public policy. We have sought to set those arguments out here. As governments prepare for economies to unfreeze (Allen et al., 2020), or prepare for future pandemics, this analysis urges policymakers to better understand the scope and limitations of policy responses available to them.

## Footnotes

1 Also see Boettke and Powell (2021, p. 1095), whose analysis includes sorting society “into two discreet groups of young/healthy and old/infirm”.

2 For instance, Leeson and Rouanet (2021, p. 1109) argue that it is also possible that behaviours that increase others’ risks of infection can confer positive externalities on those who are already voluntarily locking others away, for instance by reaching ‘herd immunity’ faster.

3 We use the term ‘dictator’ in the technical sense to refer to a government’s largely suspending normal democratic or parliamentary processes in order to impose choices made by a select insider expert group on a civilian population, which is then strictly enforced.

4 Social preferences are defined as other people’s utility functions appearing as arguments in an individual’s utility function (Fehr & Fischbacher, 2002).

## References

[CR1] Acemoglu, D., Chernozhukov, V., Werning, I., & Whinston, M. (2020). *A multi-risk sir model with optimally targeted lockdown*. NBER working papers w27102.

[CR2] Allen, D.W.E., Berg, C., Davidson, S., Lane, A.M., & Potts. J. (2020). *Unfreeze how to create a high growth economy after the pandemic*. American Institute for Economic Research.

[CR3] Allen, D. W. E., & Berg, C. (2017). Subjective political economy. *New Perspectives on Political Economy,**13*(1–2), 19–40.

[CR4] Alvarez, F., Argente, D., & Lippi, F. (2020). *A simple planning problem for COVID-19 lockdown*. NBER Working Paper w26981.

[CR5] Arrow, K. (1950). A difficulty in the concept of social welfare. *Journal of Political Economy,**58*(4), 328–346.

[CR6] Arrow, K. (1951). *Social choice and individual values*. Wiley.

[CR8] Boettke, P., & Powell, B. (2021). The political economy of the COVID-19 pandemic. *Southern Economic Journal,**87*(4), 1090–1106. 33821055 10.1002/soej.12488 PMC8014844

[CR9] Born, B., Dietrich, A., & Müller, G. (2021). The lockdown effect: A counterfactual for Sweden. *PLoS ONE, 16*(4), e0249732. 33831093 10.1371/journal.pone.0249732 PMC8031244

[CR10] Brennan, G., & Buchanan, J. (1984). Voter choice: Evaluating political alternatives. *American Behavioral Scientist,**28*, 185–201.

[CR11] Buchanan, J., & Tullock, G. (1962). *The calculus of consent*. University of Michigan Press.

[CR12] Buchanan, J. (1964). What should economists do? *Southern Economic Journal,**30*(3), 213–222.

[CR13] Buchanan, J., & Stubblebine, C. (1962). Externality. *Economica,**29*, 371–384.

[CR14] Caplan, B. (2007). *The myth of the rational voter*. Princeton University Press.

[CR15] Chang, R., & Velasco, A. (2020). Economic policy incentives to preserve lives and livelihoods. *Covid Economics,**14*, 33–56.

[CR16] Coase, R. (1960) The Problem of Social Cost. *Journal of Law and Economics*. 3: 1-44. Reproduced in R. Coase. 1988. *The Firm, the Market and the Law*. University of Chicago Press: Chicago.

[CR17] Coase, R. (1988). Notes on the problem of social cost. In R. Coase (Ed.), *The firm, the market and the law. *University of Chicago Press.

[CR18] Coase, R. (1937). The nature of the firm. *Economica,**4*, 386–405.

[CR19] Coase, R. (1959). The federal communications commission. *Journal of Law and Economics,**2*, 1–40.

[CR20] Coyne, C. J., Duncan, T. K., & Hall, A. R. (2021). The political economy of state responses to infectious disease. *Southern Economic Journal,**87*(4), 1119–1137. 33821053 10.1002/soej.12490 PMC8014786

[CR21] Demsetz, H. (1969). Information and efficiency: Another viewpoint. *Journal of Law and Economics,**12*, 1–22.

[CR22] Djankov, S., Glaeser, E., La Porta, R., Lopez-de-Silanes, F., & Shleifer, A. (2003). The new comparative economics. *Journal of Comparative Economics,**31*, 595–619.

[CR23] Downs, A. (1957). *An economic theory of democracy harper row*. Harper Row.

[CR24] Eichenbaum, M., Rebelo, S., & Trabandt, M. (2020). *The macroeconomics of epidemics*. NBER Working Papers w26882.

[CR25] Fehr, E., & Fischbacher, U. (2002). Why social preferences matter: The impact of non-selfish motives on competition, cooperation and incentives. *Economic Journal,**112*(478), 1–33.

[CR26] Fox, G. (2007). The real coase theorems. *Cato Journal,**27*(3), 373–398.

[CR27] Garibaldi, P., Moen, E., & Pissarides, C. (2020). Modelling contacts and transitions in the SIR epidemics model. *Covid Economics,**5*, 1–20.

[CR28] Garzarelli, G., Keeton, L., & Sitoe, A. A. (2022). Rights redistribution and COVID-19 lockdown policy. *European Journal of Law and Economics*. Available online. 10.1007/s10657-022-09732-x PMC8980515 35924088

[CR29] Gonzalez-Eiras, M., & Niepelt, D. (2020). On the optimal lockdown during an epidemic. *Covid Economics,**7*, 68–87.

[CR32] Jones, C., Philippon, T., & Venkateswaran, V. (2020). Optimal mitigation strategies in a pandemic: Social distancing and work from home. NBER Working Papers w26984.

[CR33] Leeson, P. T., & Rouanet, L. (2021). Externality and COVID-19. *Southern Economic Journal,**87*(4), 1107–1118. 33821050 10.1002/soej.12497 PMC8014130

[CR34] Marciano, A. (2018). ‘Why is “stigler’s Coase theorem” stiglerian? A methodological explanation. In *Including a symposium on Bruce Caldwell’s beyond positivism after 35 years.* Emerald Publishing Limited.

[CR35] McChesney, F. S. (2006). Coase, demsetz, and the unending externality debate. *Cato Journal,**26*(1), 179–200.

[CR36] McCloskey, D. (1988). The so-called coase theorem. *Eastern Economic Journal,**24*, 367–371.

[CR37] Mises, L. (1944). Bureaucracy. Republished by Mises Institute https://mises.org/library/bureaucracy.

[CR38] Mueller, D. (1976). Public choice: A survey. *Journal of Economic Literature,**14*(2), 395–433.

[CR39] Mueller, J. (1985). *War, presidents, and public opinion*. University Press of America.

[CR40] Niskanen, W. (1971). *Bureaucracy and representative government*. Transaction Publishers.

[CR41] Oslon, M. (1982). *Decline and rise of nations*. Yale University Press.

[CR42] Paniagua, P., & Rayamajhee, V. (2021). A polycentric approach for pandemic governance: Nested externalities and co-production challenges. *Journal of Institutional Economics*. 10.1017/S1744137421000795

[CR43] Pennington, M. (2021). Hayek on complexity, uncertainty and pandemic response. *The Review of Austrian Economics,**34*(2), 203–220.

[CR44] Powell, B. (2021). Government failure vs the market process during the COVID-19 pandemic. Available at SSRN 3919790.

[CR45] Rayamajhee, V., Shrestha, S., & Paniagua, P. (2021). Governing nested externalities during a pandemic: Social distancing as a coproduction problem. *Cosmos and Taxis,**9*(5–6), 64–80.

[CR46] Smith, A., Wagner, R. E., & Yandle, B. (2011). A theory of entangled political economy, with application to TARP and NRA. *Public Choice,**148*, 45–66.

[CR47] Wagner, R. E. (2016). *Politics as a peculiar business: Insights from a theory of entangled political economy*. Edward Elgar.

[CR48] Williamson, O. (1985). *The economics institutions of capitalism*. The Free Press.

[CR49] Williamson, B. (2020). Beyond COVID-19 lockdown: A coasean approach with optionality. *Economic Affairs,**40*(2), 155–161.

